# Green valorization of waste bone into a nano-hydroxyapatite adsorbent for Pb(ii) removal: synthesis, physicochemical characterization, and mechanistic insights

**DOI:** 10.1039/d6ra03066a

**Published:** 2026-07-06

**Authors:** Endrias Adane Bekele, Hailemariam Assefa Korsa, Yiene Molla Desalegn, Andarge Ayele Adem

**Affiliations:** a Faculty of Materials Science and Engineering, Jimma Institute of Technology, Jimma University Jimma Ethiopia endriasadane12@gmail.com; b School of Mechanical and Chemical Engineering, Department of Mechanical Engineering, Woldia Institute of Technology, Woldia University Woldia Ethiopia; c School of Mechanical and Automotive Engineering, Department of Mechanical Engineering, College of Engineering and Technology, Dilla University Dilla Ethiopia

## Abstract

Lead (Pb(ii)) exposure poses significant health risks to various organ systems, including the hematological, renal, neurological, and cardiovascular systems. In this study, nano-hydroxyapatite (nano-HAp) was prepared *via* an ultrasonication-assisted hydrothermal method to remove Pb(ii) from an aqueous solution. The nano-HAp adsorbent was characterized using X-ray diffraction (XRD), Fourier transform infrared (FTIR) spectroscopy, scanning electron microscopy (SEM), Brunauer–Emmett–Teller (BET) surface area, and point of zero charge (pH_pzc_) analyses. The effects of various factors, including pH (2–8), initial Pb(ii) concentration (40–200 mg L^−1^), adsorbent dose (0.01–0.07 g L^−1^), and contact time (30–130 min), were investigated, with the residual Pb(ii) concentrations measured *via* atomic absorption spectroscopy (AAS). The highest removal efficiency of 99.84% was achieved at an optimum pH of 5, an initial Pb(ii) concentration of 40 mg L^−1^, an adsorbent dose of 0.03 g L^−1^, and a contact time of 110 min. Based on the coefficients of determination (*R*^2^), the pseudo-second-order kinetic (*R*^2^ = 0.9912) and Langmuir isotherm (*R*^2^ = 0.9993) models best described the adsorption process. The maximum monolayer adsorption capacity was determined to be 133.33 mg g^−1^. Thermodynamic analysis confirmed that the process was spontaneous and endothermic, as indicated by positive Δ*H* and negative Δ*G* values. Furthermore, reusability studies demonestrated that the nano-HAp adsorbent maintained a removal efficiency above 86% after five successive cycles. Therefore, the prepared nano-HAp adsorbent represents a promising, eco-friendly adsorbent for Pb(ii) removal.

## Abstract 2

Lead (Pb(ii)) exposure poses significant health risks to various organ systems, including the hematological, renal, neurological, and cardiovascular systems.

## Graphical abstract

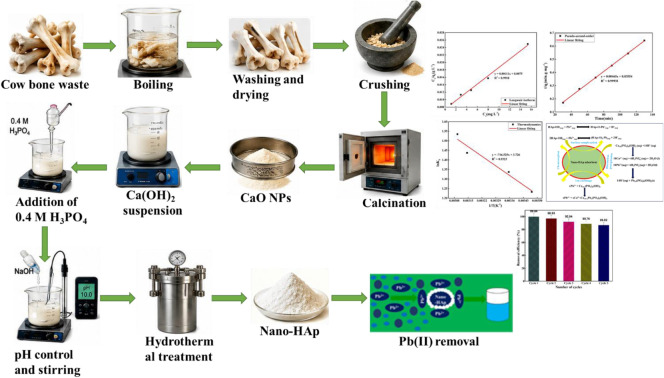

## 1. Introduction

Water is necessary for life on Earth, yet millions experience a daily lack of this vital resource.^1^ The disposal of industrial effluents and wastewater presents substantial issues due to their hazardous components, including heavy metal ions and dyes, which are major contributors to the contamination of rivers, lakes, and underground water.^2^ Heavy-metal contamination in aquatic habitats poses a significant global environmental concern due to the persistence, toxicity, and propensity for bioaccumulation.^3^ Heavy-metal pollution primarily originates from industrial waste produced by battery manufacturing, mining, electroplating, and oil refining.^4^ The heavy metal ions commonly found in environmental matrices include Cu(ii), Ni(ii), Pb(ii), Zn(ii), Cd(ii), and Hg(ii).^5^ Heavy metals, including zinc, copper, and selenium, are dense elements (≥5 g cm^−3^) that serve as essential micronutrients for plants and are important for human metabolism. However, metals such as cadmium, mercury, chromium, arsenic, thallium, and lead can be toxic, even at low concentrations.^6^

Pb(ii), heavy metal ions found in mine wastewater, are prevalent environmental pollutants that accumulate and pose toxicity risks.^7^ Pb(ii) is a toxic heavy metal that can accumulate in the human body, causing severe health problems, like brain and kidney damage, as well as central nervous system dysfunction, even in low concentrations.^8^ The World Health Organization (WHO) specifies the maximum permitted concentration of lead (Pb(ii)) in drinking water at 0.05 mg L^−1^, while the permissible values in wastewater range from 0.05 to 0.10 mg L^−1^ before discharge.^9^ Pb(ii) must be eliminated from water bodies because they are often present in the +2 and +4 valence states, can bind to biomolecules, and disrupt normal biochemical and physiological processes.^10^

Several strategies for eliminating Pb(ii) from contaminated water include ion exchange, membrane filtration, electrolysis, coagulation, and flotation. However, these methods are often costly and require high concentrations of heavy metals for effective removal.^11^ Adsorption is a promising technology for removing Pb(ii) from wastewater due to its cost-effectiveness, simplicity, potential for regeneration with an eluent solution, and reusability with minimal sludge generation.^12,13^ Selecting an adsorbent necessitates evaluating physicochemical properties, including surface area, porosity, ion-exchange capacity, and affinity for pollutants.^14,15^ Nowadays, natural and bio-derived materials are increasingly recognized as environmentally friendly and sustainable options for adsorbents.^16^ Common adsorbents include activated carbons, zeolites, clays, graphene, biomass, metal-oxide-based nanomaterials, metal–organic frameworks (MOFs), magnetic nanoparticles, industrial and farming residues, and polymeric materials.^17,18^

Sustainability and the circular economy are supported by waste-based adsorbents in wastewater treatment, transforming waste into valuable resources, lowering disposal costs, and providing a cost-effective alternative to conventional adsorbents.^19–21^ Animal-derived waste is a promising food waste type due to its high mineral content, comprising 65–70% minerals (mainly calcium and phosphorus) and 30–35% organic matter, with up to 90% being collagen protein. ^22^

One way to reduce waste and create a cost-effective adsorption system is by transforming waste materials, such as collagen, hydroxyapatite (HAp), gelatin, charcoal, and calcium phosphate, into useful adsorbents.^23^ HAp, a calcium phosphate compound with a Ca_10_(PO_4_)_6_(OH)_2_ formula, demonstrates significant potential for metal-ion sequestration due to its ion-exchange capacity, high surface reactivity, and biocompatibility.^16^ HAp is an effective adsorbent found in bones and teeth, notable for its high specific surface area and favorable chemical and physical properties that facilitate the adsorption of heavy metals.^24^ HAp has a hexagonal crystal structure (space group *P*6_3_/*m*). The unit cell contains 10 calcium cations, with four at M1 (Ca1) sites aligned in a column and surrounded by nine oxygen atoms, and six at M2 (Ca2) sites positioned at the apexes of staggered equilateral triangles, each surrounded by seven oxygen atoms.^25^ The crystalline structure of HAp provides it with exceptional qualities, such as high biocompatibility and bioactivity, non-toxicity, ease of access, cost-effectiveness, and low solubility.^26^

The preparation of HAp can be performed using various methods, such as extraction from natural sources, like bovine bones, chemical precipitation, hydrothermal, sol–gel, mechanochemical, electrochemical, combustion in solution, pyrolysis, microemulsion, and microwave-assisted hydrothermal synthesis methods.^27,28^ Ya-Wen Lin *et al.* reported the hydrothermal synthesis of eco-HAp derived from limestone sludge for Cu(ii) adsorption, with an adsorption capacity of 210 mg g^−1^.^29^ Similarly, Ling Shi *et al.* studied the use of natural HAp powder from pig-bone waste (pHAp) for rapidly adsorbing Cu(ii) in water. They found that optimal conditions for Cu(ii) (50 mg L^−1^) occurred at pH 7 and 318.15 K, achieving a maximum adsorption capacity of 50.25 mg g^−1^.^30^ Furthermore, Guo Liu *et al.* developed HAp-attapulgite composites using the co-precipitation method to effectively adsorb Cd(ii) from aqueous solutions.^31^ In another study, Yiping Su *et al.* synthesized two-dimensional holey HAp nanosheets using a microwave-assisted hydrothermal method, achieving a specific surface area of 92.9 m^2^ g^−1^. These nanosheets effectively adsorbed heavy metals, with maximum capacities of 210.5 mg g^−1^ for Pb(ii), 31.6 mg g^−1^ for Cu(ii), and 24.9 mg g^−1^ for Cd(ii).^32^ In addition, Huawei Wang *et al.* successfully synthesized biogenic HAp from chicken waste, achieving over 99% removal efficiency of Pb(ii) from wastewater. Optimal conditions included an initial pH of 3.0, an initial Pb(ii) concentration of 208 mg L^−1^, and an adsorbent dosage of 1 g L^−1^.^33^ Notably, Zongqiang Zhu *et al.* demonstrated that strontium-doped HAp (Sr-HAp), produced by the sol–gel method, effectively removed Pb(ii) from water, achieving an adsorption capacity of 651.175 mg g^−1^, which was significantly higher than that of HAp.^34^

Discarded animal bones represent a valuable precursor for preparing eco-friendly nano-adsorbents, yet further research is required to optimize their application for Pb(ii) removal. Therefore, this work aims to prepare a nano-HAp adsorbent *via* an ultrasonication-assisted hydrothermal method and evaluate its adsorptive performance for Pb(ii). The prepared nano-HAp adsorbent was characterized using X-ray diffraction (XRD), Fourier transform infrared (FTIR) spectroscopy, scanning electron microscopy (SEM), Brunauer–Emmett–Teller (BET) surface area, and point of zero charge (pH_pzc_) analyses. Batch adsorption experiments were conducted to evaluate the effects of pH, initial Pb(ii) concentration, adsorbent dose, and contact time, with the residual Pb(ii) concentrations quantified *via* atomic absorption spectroscopy (AAS). Furthermore, isotherm, kinetic, and thermodynamic modelling were investigated to elucidate the underlying adsorption mechanisms. Finally, the reusability potential of the nano-HAp adsorbent was examined in detail.

## 2. Materials and methods

### 2.1. Materials and chemicals

The study utilized cow bone waste collected from the fresh discard of the Butcher shop in Jimma, Ethiopia. Lead(ii) nitrate [Pb(NO_3_)_2_, 99%], sodium hydroxide pellet (NaOH, 98%), potassium bromide pellet (KBr, 99%), potassium hydroxide (KOH, 99%), ethanol (C_2_H_5_OH, 99.9%), hydrochloric acid (HCl, 37.0%) and phosphoric acid (H_3_PO_4_, 85%) were purchased from Sigma-Aldrich and Merck Ltd. All chemicals were used without further purification, and distilled water was employed throughout the process.

### 2.2. Adsorbent preparation

#### 2.2.1. Preparation of nano-HAp

The preparation of nano-hydroxyapatite (nano-HAp) from cow bone waste was conducted using a modified version of a previously reported method.^35^ Initially, cow bone waste was boiled in distilled water for 6 h to remove fats and contaminants. The bones were then thoroughly cleaned of residual flesh and soft tissues, followed by drying in an oven at 80 °C for 12 h. The dried bones were crushed into small pieces using a hammer, and then, 15 g of the resulting powder was placed in a silica crucible and calcined at 900 °C for 3 h. The resulting calcium oxide (CaO) nanoparticles were sieved to a size below 63 µm and dispersed in 100 mL of distilled water to form a calcium hydroxide (Ca(OH)_2_) suspension, which was sonicated for 4 h. Thereafter, 0.4 M phosphoric acid (H_3_PO_4_) was gradually added to the mixture while keeping the pH around 10 with sodium hydroxide (NaOH), and the solution was stirred continuously for 2 h to achieve homogeneity. The white precipitate solution was heated at 180 °C for 24 h in a stainless-steel autoclave to ensure complete precipitation. Finally, the precipitate was calcined at 900 °C for 2 h to prepare the nano-HAp adsorbent.

### 2.3. Zero point of charge (pH_pzc_)

The pH at which the nano-HAp-adsorbent surface becomes electrically neutral is referred to as the point of zero charge (pH_pzc_). pH_pzc_ of the nano-HAp adsorbent was found using the solid addition method with a 0.04 M NaCl solution.^36^ In this method, 20 mL of 0.04 M NaCl solution was added to several conical flasks. The initial pH (pH_i_) of each solution was adjusted from 2 to 10 using HCl and NaOH, followed by the addition of 0.03 g of nano-HAp adsorbent, with the suspensions stirred for 24 h at room temperature. After equilibration, the final pH (pH_f_) was measured. The change in pH (ΔpH) was determined as ΔpH = pH_i_–pH_f_ and plotted against the initial pH (pH_i_). The pH_pzc_ value corresponds to the point where the curve intersects the abscissa, indicating ΔpH = 0.

### 2.4. Materials characterization

The physicochemical characteristics of the prepared adsorbents were characterized by advanced techniques. X-ray diffraction (Drawell XRD-700, China) was conducted to verify the crystalline phase and structural integrity of the adsorbent using Cu Kα1 (*λ* = 1.5046 Å) under 30 kV and 25 mA operating conditions, with the scanning angle (2*θ*) range of 10°–80° with a scanning rate of 0.03° min^−1^. The crystallite sizes of the adsorbents were evaluated from the peak broadening of the XRD patterns based on Scherrer's eqn (1) as follows:^37^1
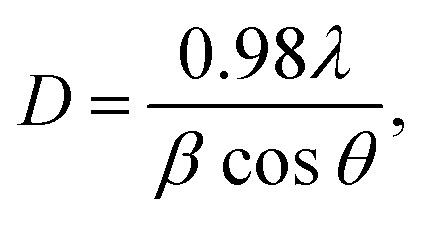
where *D* is the crystallite size (nm), *λ* is the wavelength of the monochromatic X-ray beam (*λ* = 0.154056 nm for Cu Kα radiation), *β* is the full width at half-maximum for the diffraction peak under consideration (rad) and *θ* is the diffraction angle (degrees). Fourier transform infrared spectroscopy (FTIR, Spectrum Two, PerkinElmer, USA), in the wavenumber range of 400–4000 cm^−1^, was used to identify the functional groups. The surface morphology was determined by scanning electron microscopy (SEM, JSM-7500F, JEOL, Japan) at an accelerating voltage of 15 kV. The equilibrium concentrations of Pb(ii) were analyzed *via* an atomic absorption spectrophotometer (model Thermo Scientific ICE 3000 series). The surface area, average pore size, and total pore volume of the nano-HAp adsorbent were measured using Brunauer–Emmett–Teller (BET) nitrogen adsorption/desorption isotherms at 77 K with a high-speed gas sorption analyzer (Nova 4200e Quantachrome Instruments, Boyton Beach, FL, USA).

### 2.5. Adsorption experiment

Batch adsorption studies investigated the effectiveness of the nano-HAp adsorbent for adsorbing Pb(ii). A 1000 mg L^−1^ Pb(ii) standard stock solution was created by dissolving 1 g of Pb(NO_3_)_2_ in distilled water within a 1000-mL volumetric flask. In a 250-mL volumetric flask, 100 mL of 40 mg L^−1^ Pb(ii) solution was mixed with 0.03 g L^−1^ of adsorbent. For 24 h at room temperature, the flask was shaken at a constant 200 rpm on an orbital shaker. By adjusting operating parameters, such as pH (2–8), initial Pb(ii) concentration (40–200 mg L^−1^), adsorbent dose (0.01–0.07 g L^−1^), and contact time (30–130 min), the adsorption of Pb(ii) onto the nano-HAp adsorbent was optimized. The experimental pH was adjusted dropwise using a 0.1 M NaOH/HCl solution. The samples were taken out of the shaker after equilibrium was reached, and the liquid supernatant was separated using a centrifuge. Atomic absorption spectroscopy was used to determine each sample's residual Pb(ii) content. To get an average value, each adsorption experiment was carried out 3 times.

The percentage removal of Pb(ii) is determined using eqn (2) as follows:^38^2
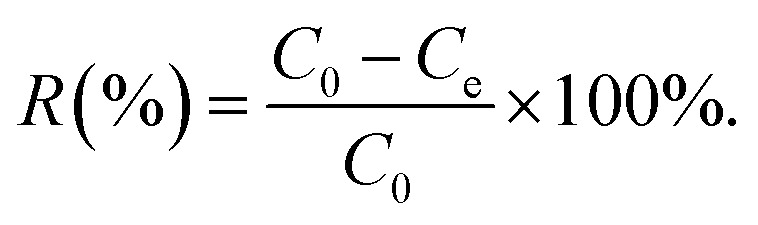


The amount of Pb(ii) adsorbed per unit mass of nano-HAp adsorbent at equilibrium (*q*_e_) and at different time intervals (*t*), (*q*_t_) were calculated using eqn (3) and (4), respectively:^17,39^3
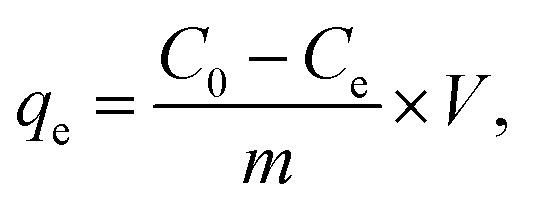
4
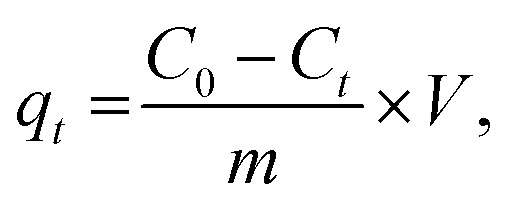
where *q*_e_ is the amount of adsorbate per mass of the adsorbent (mg g^−1^), *q*_*t*_ is the adsorption capacity (mg g^−1^), and *R* is the removal efficiency (%). *C*_0_, *C*_e_, and *C*_*t*_ are the initial, equilibrium and at-time-t Pb(ii) concentrations (mg L^−1^), respectively; *V* is the volume of solution (L); and *m* is the mass of the adsorbent (g L^−1^).

### 2.6. Adsorption isotherms

Both the chemical interaction between the adsorbent and the adsorbate, along with the adsorption strength, were evaluated using isotherms. This study selected the most suitable model for predicting the adsorption of Pb(ii) onto the nano-HAp adsorbent by testing isotherm models: the Freundlich, Langmuir, Temkin, and Dubinin–Radushkevich (D–R).^22^

In the monolayer adsorption process involving heterogeneous surface energy, the Freundlich isotherm is based on the interactions among adsorbed species. The linear form of this model is expressed by eqn (5) as follows:5
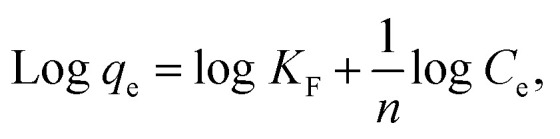
where *K*_F_ is the Freundlich constant, which implies the affinity of the heterogeneous surface towards the adsorbent and *n*^−1^ verifies the favorability of the adsorption process.

The Langmuir isotherm model, which may be represented using eqn (6), implies that adsorption takes place on homogeneous surfaces without contact between the deposited Pb(ii) as follows:6
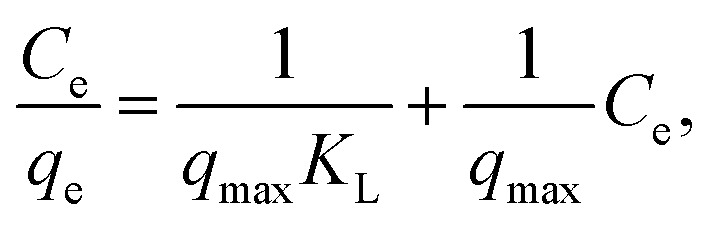
where *q*_max_ (mg g^−1^) is the monolayer adsorption capacity of the adsorbent, and *K*_L_ (L mg^−1^) is the Langmuir adsorption constant, which represents the affinity of the binding site. A straight line with a slope (*q*_max_^−1^) and an intercept (*K*_L_^−1^*q*_max_^−1^) is produced when *C*_e_ is plotted against 
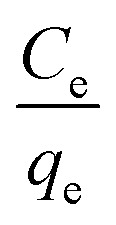
. The slope can be divided by the intercept to determine the Langmuir constants (*K*_L_ and *q*_max_).

Eqn (7) computes the dimensionless separation factor equilibrium parameter, *K*_L_, which yields the expected adsorption efficiency of the process as follows:7
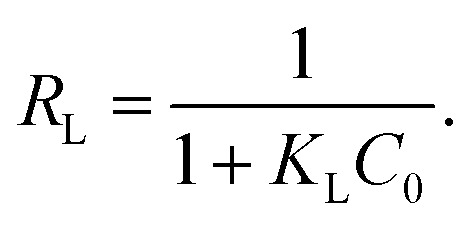


The *R*_L_ values present four possibilities: favorable (0 < *R*_L_ < 1), unfavorable (*R*_L_ > 1), irreversible (*R*_L_ = 0), or linear (*R*_L_ = 1).

According to the Temkin isotherm model, the heat of adsorption will decrease linearly with the age of the surface cover that arises from the interaction between the adsorbent and the adsorbate. The linearized form is expressed in eqn (8) as follows:^40^

8 *q*_e_ = *b*_T_ ln *A*_T_ + *b*_T_ ln *C*_e_,

where 
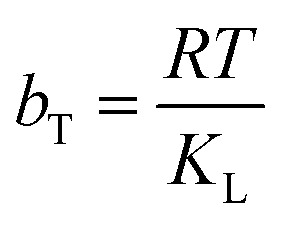
, *b*_T_ is the Temkin isotherm constant related to the heat of adsorption (J mol^−1^), *A*_T_ is the Temkin isotherm equilibrium binding constant (L g^−1^), *T* is the absolute temperature (K), and *R* is the universal gas constant (8.314 J mol^−1^ K^−1^).

Experiments in a thermostatic shaker were conducted at 200 rpm until equilibrium was reached for isotherm studies. At 298 K, tests were conducted using 100 mL of initial Pb(ii) concentrations of 40, 80, 120, 160, and 200 mg L^−1^.

The D–R isotherm distinguishes between physical and chemical adsorption mechanisms for Pb(ii), as outlined in eqn (9) as follows:^41^

9 *q*_e_ = *q*_m_ exp(−*βε*^2^),

where *q*_e_ (mol g^−1^) is the amount of metal adsorbed per unit mass of adsorbent, *q*_m_ (mol g^−1^) is the monolayer adsorption capacity, *β* (mol^2^ kJ^−2^) is the activity coefficient related to the mean sorption energy, and *ε* is the Polanyi potential and can be calculated using eqn (10) as follows:10
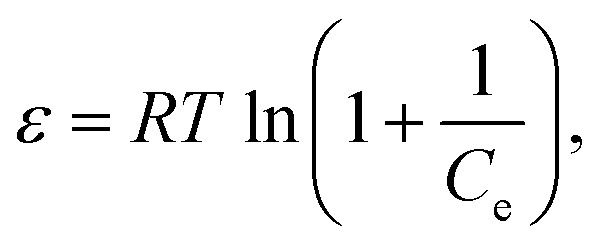
where *C*_e_ (mol L^−1^) is the equilibrium Pb(ii) concentration in an aqueous solution. The mean adsorption energy, *E* (kJ mol^−1^), can be calculated using eqn (11) as follows:11
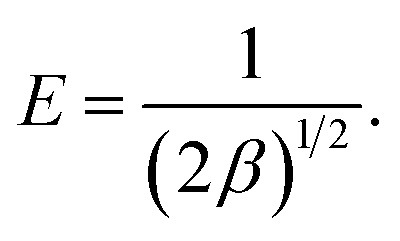


If the energy (*E*) is between 8 and 16 kJ mol^−1^, it shows chemisorption; if below 8 kJ mol^−1^, it suggests a physical process.^42^

The linear form of the D–R isotherm model is expressed as eqn (12) as follows:

12 ln *q*_e_ = ln *q*_m_ − *βε*^2^.

The D–R model constants, *q*_m_ and *β*, can be determined from the intercept and slope of the linear plot of ln *q*_e_*versus ε*^2^, respectively.

### 2.7. Adsorption kinetics

Nano-HAp adsorbent data on Pb(ii) adsorption were analyzed to understand the kinetic behavior of the adsorption process from aqueous solutions.^43^ This study investigated how varying contact time (30–130 min) affects the adsorption efficiency of Pb(ii) at a fixed volume of 100 mL, focusing on the kinetics of the adsorption process. Four kinetics models, pseudo-first-order (PFO), pseudo-second-order (PSO), intra-particle diffusion (IPD), and double constant equation (DCE), were utilized to clarify the adsorption mechanism. Eqn (13)–(16) were used to find the linear version for four different model orders related to the nano-HAp adsorbent as follows:^44,45^

13 ln(*q*_e_ − *q*_t_) = ln(*q*_e_) − *k*_1_*t*,

14
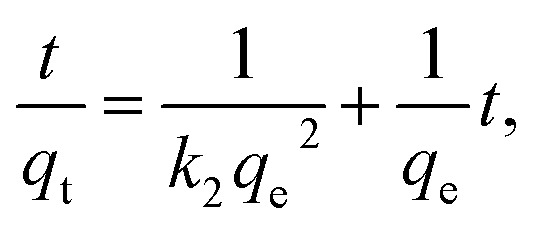

15 *q*_t_ = *C* + *K*_i_*t*^1/2^,

16 ln *q*_t_ = ln *A* + *B* ln *t*,

where *q*_e_ (mg g^−1^) and *q*_t_ (mg g^−1^) are the adsorption capacity at equilibrium and time *t* (min), respectively and *C* is the intercept, which is related to the boundary layer thickness (mmol g^−1^). *k*_1_ (min^−1^), *k*_2_ (g mg^−1^ min^−1^), and *K*_i_ (mg g^−1^ min^−1/2^) are the rate constants of the pseudo first-order, pseudo-second-order, and intraparticle diffusion models. *A* and *B* are the double-constant equation model constants.

### 2.8. Adsorption thermodynamics

To understand the impact of temperature on adsorption, thermodynamic parameters, such as enthalpy changes (Δ*H*), entropy changes (Δ*S*), and Gibbs free energy change (Δ*G*), were analyzed. Gibbs free energy determines spontaneity (Δ*G* < 0 indicates a spontaneous process), while enthalpy values indicate whether the process is endothermic (Δ*H* > 0) or exothermic (Δ*H* < 0). Higher adsorption performance results from positive values of Δ*S*, which signify relatively high randomness at the solid–liquid interphase during the sorption processes.^46^ Van't Hoff eqn (17)–(19) were used to calculate these thermodynamic characteristics as follows:

17 Δ*G* = −*RT* ln *K*_F_,

18
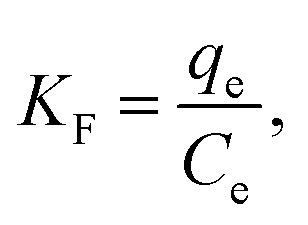
19
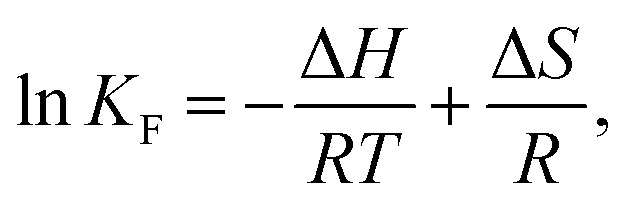
where *K*_F_ represented the adsorption equilibrium constant (L g^−1^), *R* is the gas constant (8.314 J mol^−1^ K^−1^), and *T* is the thermodynamic reactive temperature (K). Δ*G* is the Gibbs free energy (kJ mol^−1^); Δ*H* (kJ mol^−1^) and Δ*S* (J mol^−1^ K^−1^) represent the enthalpy change and the entropy change, respectively. Adsorption experiments were conducted at four different temperatures (298–323 K).

### 2.9. Reusability experiments

In the regeneration experiment, the optimal conditions selected were a pH of 5 for the desorption solution, an initial Pb(ii) concentration of 40 mg L^−1^, an adsorbent dose of 0.03 g L^−1^, and a contact time of 110 min for the nano-HAp adsorbent reusability. The nano-HAp adsorbent was dried at 80 °C after washing away the desorption solution. The experiment involved five adsorption–desorption cycles conducted in triplicate to evaluate the robustness of the adsorbent, focusing on its performance and efficiency in removing Pb(ii) during each regeneration.

## 3. Results and discussions

### 3.1. XRD analysis

**Fig. 1:**
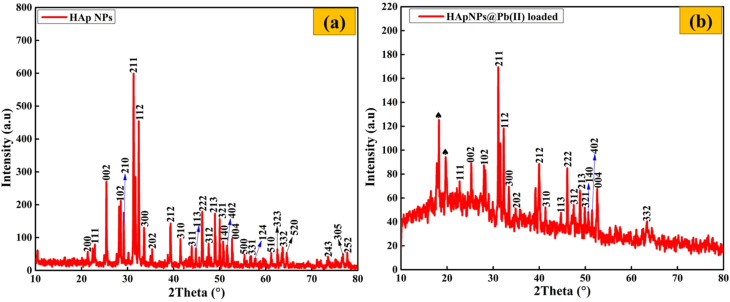
XRD patterns of the nano-HAp adsorbent (a) before and (b) after Pb(ii) adsorption.

The XRD patterns of the nano-HAp adsorbent before and after adsorption are depicted in Fig. 1a and b, respectively. The XRD pattern of the nano-HAp adsorbent before adsorption is displayed in Fig. 1a. The (200), (111), (002), (102), (210), (211),(112), (300), (202), (212), (310), (311), (113), (222), (312), (213), (321), (140), (402), (004), (500), (331), (124), (510), (323), (332), (520), (243), (305), and (252) lattice planes correspond to sharp and strong XRD peaks observed at 2*θ* values of 21.23°, 22.74°, 25.35°, 28.49°, 29.32°, 31.37°, 32.47°, 33.56°, 35.35°, 39.32°, 41.51°, 43.98°, 44.98°, 46.17°, 47.54°, 49.05°, 50.62°, 50.84°, 51.66°, 52.61°, 55.35°, 56.73°, 57.68°, 61.24°, 62.49°, 63.72°, 64.54°, 73.58°, 76.73°, and 77.70°.^47^ Standard JCPDS No. 09-0432 confirmed the verification of the obtained nano-HAp adsorbent.^48^Fig. 1b displays the XRD pattern of the nano-HAp adsorbent after adsorption. The adhesion of Pb(ii) to the nano-HAp adsorbent caused two new strong peaks at 2*θ* values of 18.21° and 19.72°. Moreover, minor peak shifts from 22.74°–22.60°, 25.35°–25.21°, 28.49°–28.09°, 31.37°–31.10°, 32.47°–32.19°, 33.56°–33.44°, 35.35°–34.95°, 39.32°–40.02°, 44.95°–44.67°, 46.17°–46.03°, 47.54°–47.40°, 49.05°–48.91°, 50.62°–49.88°, 50.84°–50.56°, 51.66°–51.38°, and 63.72°–63.31° further validated the adsorption of Pb(ii) on the nano-HAp adsorbent surface. Additionally, the peaks at 2*θ* values of 29.32°, 43.98°, 55.35°, 56.73°, 57.68°, 61.24°, 62.49°, 64.54°, 73.58°, 76.73°, and 77.70° (252) vanished upon adsorption, indicating that Pb(ii) was successfully adsorbed. The prepared nano-HAp adsorbent had crystallite sizes of 15.56 nm before and 18.64 nm after adsorption.

### 3.2. FTIR analysis

**Fig. 2:**
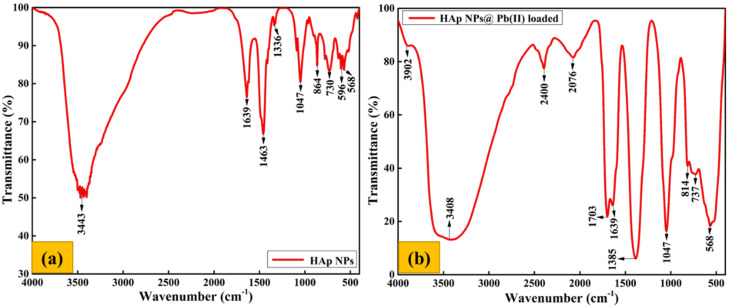
FTIR spectra of the nano-HAp adsorbent (a) before and (b) after Pb(ii) adsorption.

The FTIR spectra of the nano-HAp adsorbent before and after Pb(ii) adsorption are shown in Fig. 2a and b, respectively. Fig. 2a depicts the FTIR spectra of the nano-HAp adsorbent before adsorption. The absorption band at 1047 cm^−1^ corresponded to the PO_4_^3−^ group, associated with the asymmetrical stretching of the P–O bond. The peak at 568 cm^−1^ to 596 cm^−1^ corresponded to the asymmetric stretching of the O–P–O bond.^49–51^ The peaks at 1463 cm^−1^, 1336 cm^−1^, and 864 cm^−1^ were ascribed to the asymmetric stretching and bending vibrations of PO_4_^3−^ and/or characterized by the substitution ofPO_4_^3−^ with CO_3_^2−^ during synthesis.^52^ The O–H stretching and bending vibrations of water molecules were associated with peaks at 3443 cm^−1^ and 1639 cm^−1^.^53,54^ The band at 730 cm^−1^ was associated with the bending vibration mode of the O–H group in the nano-HAp adsorbent.^55^ The FTIR spectra of the nano-HAp adsorbent after Pb(ii) adsorption are depicted in Fig. 2b. The appearance of new peaks after Pb(ii) adsorption indicated the interaction of Pb(ii) and the surface functional groups of the nano-HAp adsorbent. The broad and intense band at 3408 cm^−1^ and the weak band at 3902 cm^−1^ corresponded to the O–H stretching vibrations of surface hydroxyl groups and adsorbed water molecules. The peak around 1703 cm^−1^ was attributed to the C=O stretching vibration, while the peaks observed near 2400 cm^−1^ and 2076 cm^−1^ may be associated with atmospheric CO_2_ adsorption, CO_3_^−^and surface complexation after Pb(ii) adsorption. The asymmetric stretching vibration (CO_3_^−^) was identified as the cause of the peaks discovered at 1385 cm^−1^. Moreover, Pb(ii) adsorbed on the nano-HAp adsorbent surface was confirmed by peak shifts seen from 864 cm^−1^–814 cm^−1^ to 730 cm^−1^–737 cm^−1^ and the disappearance of 1463 cm^−1^ and 596 cm^−1^. Another indication of the Pb(ii) adsorption was the distinct and strong peaks for the carbonate and phosphate groups, which were followed by the hydroxyl group.

### 3.3. SEM analysis

**Fig. 3:**
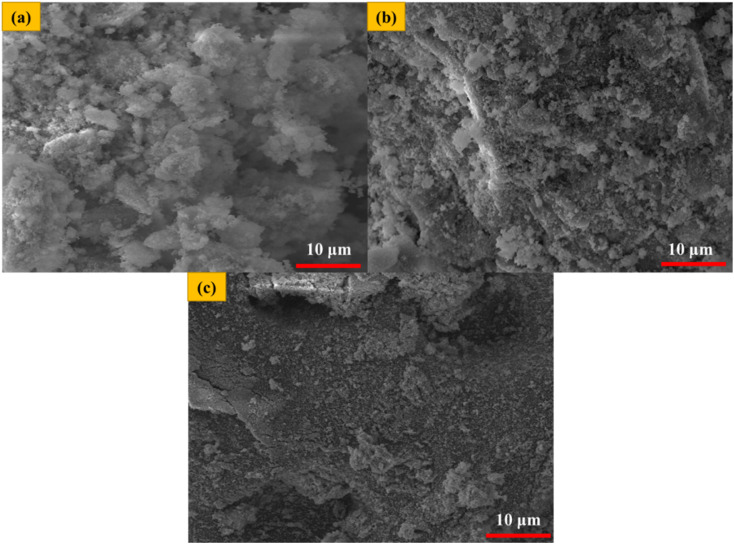
SEM images of the nano-HAp adsorbent before adsorption under magnifications of (a) 5 k× and (b) 30 k× and (c) after Pb(ii) adsorption.

The surface shape and microstructure of the nano-HAp adsorbent, both before and after adsorption, are displayed in Fig. 3a–c. The SEM picture of the nano-HAp adsorbent at a 5× magnification is shown in Fig. 3a. The illustration displayed a pore structure made up of aggregated amorphous particles with an uneven form. Fig. 3b displays the SEM image of the nano-HAp adsorbent at a 30× magnification. The picture showed semi-spherical crystals that were rough and evenly spaced. The porous nature of the nanoparticle adsorbent was further demonstrated by the many voids and pores shown in the figure. The adsorption of Pb(ii) was facilitated by the increased surface roughness, increased pore count, and increased exposure of adsorption sites. The nano-HAp adsorbent surface looked smooth and tightly packed during adsorption, as seen in Fig. 3c. This could be due to the presence of Pb(ii) on the adsorbent surface.

### 3.4. BET analysis

**Fig. 4:**
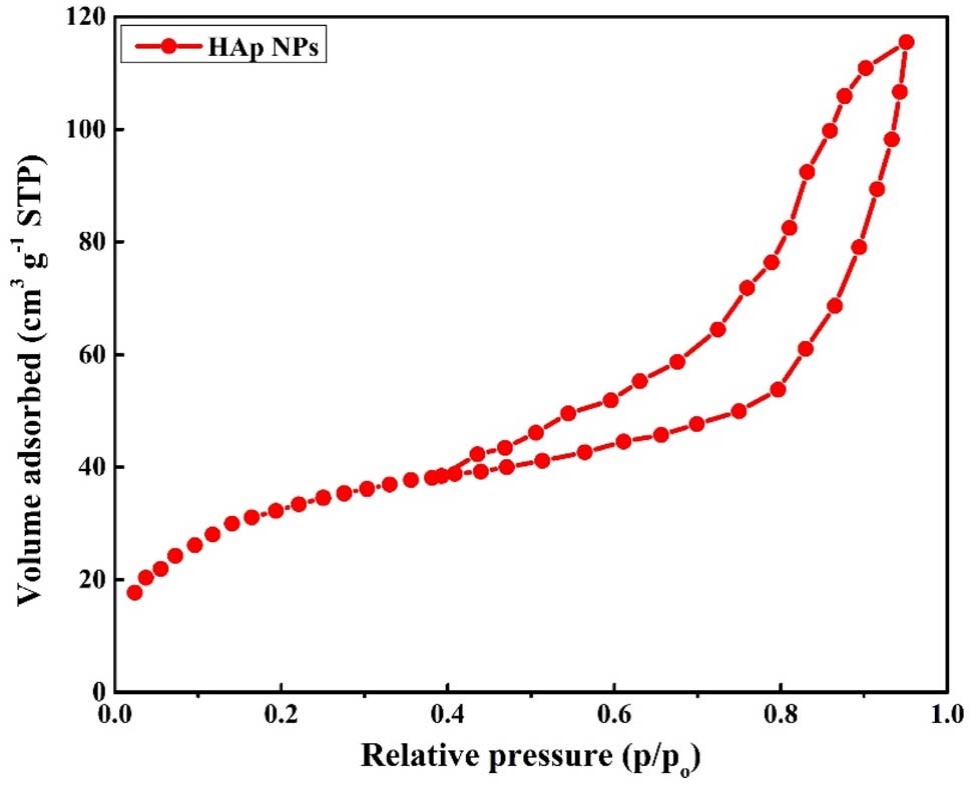
N_2_ adsorption–desorption isotherms of the nano-HAp adsorbent.

Using the BET method in a nitrogen (N_2_) atmosphere, the surface area of the prepared nano-HAp adsorbent was examined in relation to N_2_ adsorption–desorption isotherms. The obtained isotherm was found to belong to type IV classification in the IUPAC system with a H3-type hysteresis loop, as shown by a broad isotherm curve seen in Fig. 4. The BET surface area of the nano-HAp adsorbent was determined to be 98.8 m^2^ g^−1^. Furthermore, the average adsorption pore width was 0.680 nm, and the total pore volume was 0.419 m^3^ g^−1^.

### 3.5. Zero point of charge (pH_pzc_)

**Fig. 5:**
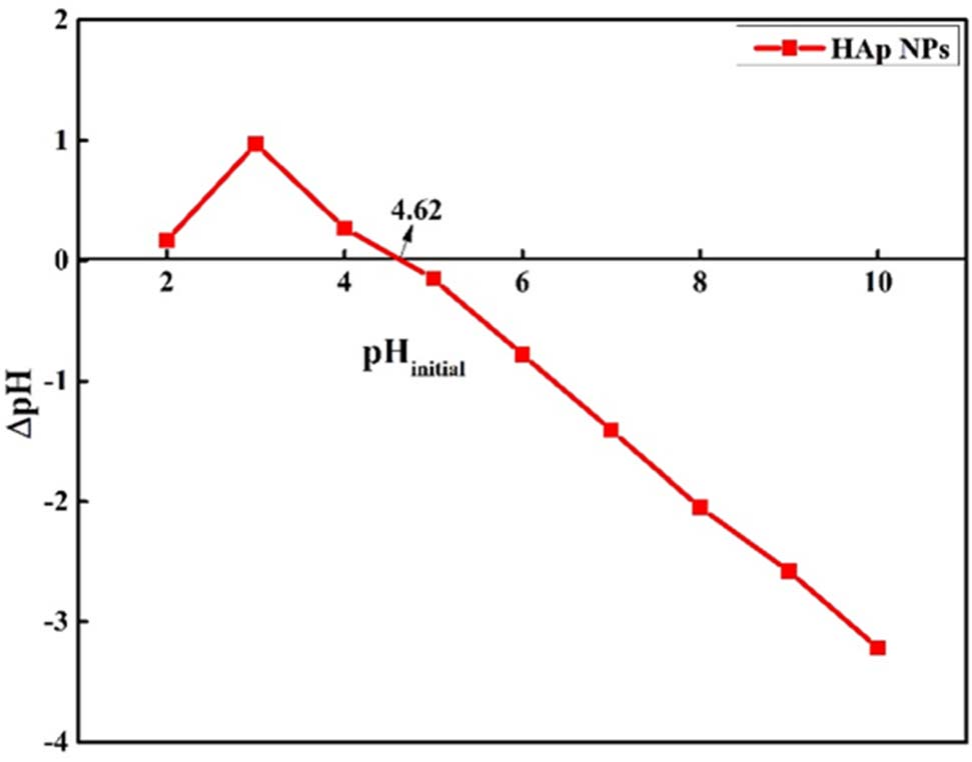
Change in the pH (ΔpH) of the nano-HAp adsorbent at different pH values.

The investigation of the point of zero charge (pH_pzc_) provided crucial information on how the electrostatic properties of the nano-HAp adsorbent surface were influenced by the pH level of the solutions. The pH_pzc_ is the point where pH_final_–pH_initial_ (ΔpH) *versus* pH_initial_ is zero. The results of pH_pzc_ for the nano-HAp adsorbent are shown in Fig. 5 and found to be 4.62. At a pH > pH_pzc_, the surface charge of the adsorbent was negative, which was favorable for the adsorption of cationic species, and at pH < pH_pzc_, the surface charge on the adsorbent was a net positive charge, which favored the adsorption of anions.

### 3.6. Adsorption parameters

#### 3.6.1. Effect of pH

**Fig. 6:**
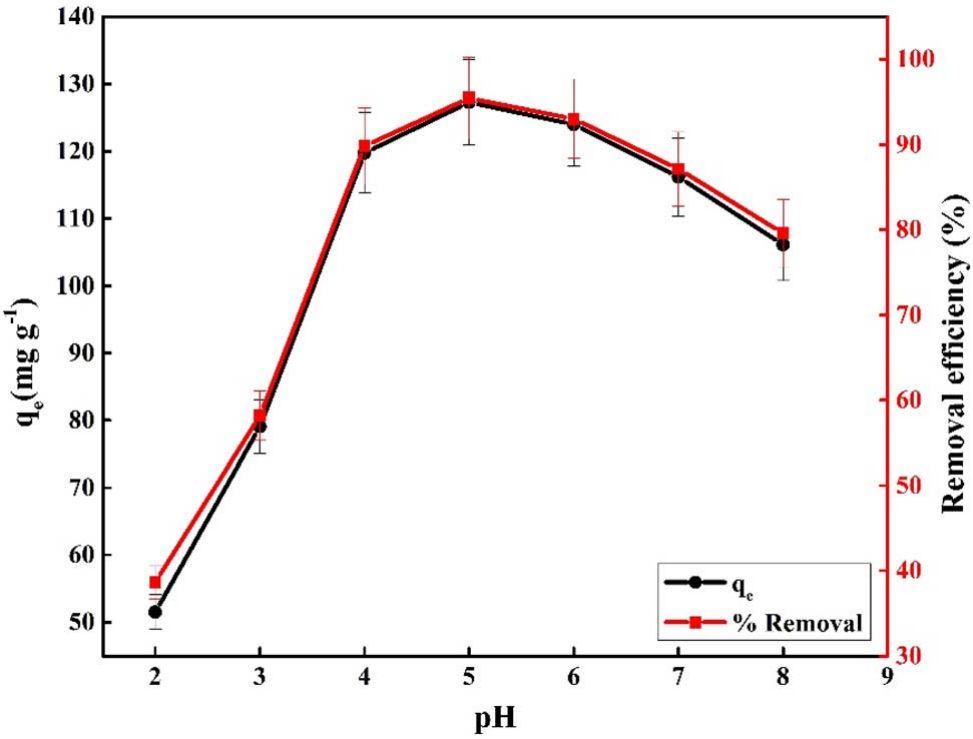
Effect of pH on the adsorption efficiency of Pb(ii) by the nano-HAp adsorbent.

pH significantly affects the adsorption process by changing the surface charges of the adsorbent and adsorbate. The impact of pH on Pb(ii) adsorption effectiveness is seen in Fig. 6. The impact of pH was assessed between 2 and 9 while maintaining an initial Pb(ii) concentration of 40 mg L^−1^, an adsorbent dose of 0.03 g L^−1^, and a contact period of 110 min. The result showed that raising the pH from 2 to 5 enhanced the removal efficiency from 38.64% to 96.48%. However, as pH rose from 5 to 9, adsorption capabilities decreased by 79.38%. These findings suggested that there was less competition with Pb(ii) for binding sites on the adsorbent's surface as a result of the drop in H^+^ ion concentration in acidic solutions. Low adsorption efficiency was caused by large amounts of H^+^ ions occupying binding sites at pH < 4.62, which hindered Pb(ii) from effectively reaching the adsorption surface. Furthermore, Pb^2+^, PbOH^+^, and Pb_2_(OH)_3_^+^ were the primary species of Pb(ii) at pH < 4.62. This resulted in a decrease in the electrostatic interaction between Pb(ii) ions and the HAp nanoparticle adsorbent. The formation of Pb(ii) hydroxide complexes and lead hydroxide (Pb(OH)_2_) precipitates at pH > 4.62 suggested that the optimal pH range for the nano-HAp adsorbent to adsorb Pb(ii) was between 5 and 9.

#### 3.6.2. Effect of Pb(ii) initial concentration

**Fig. 7:**
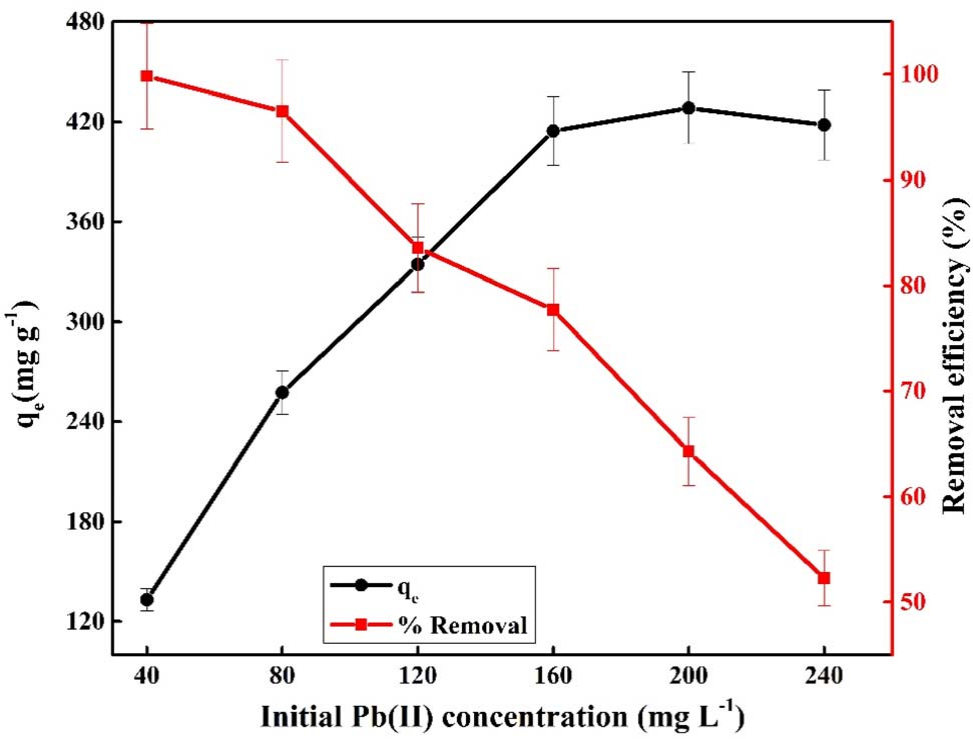
Effect of the initial Pb(ii) concentration on the removal efficiency of Pb(ii) by the nano-HAp adsorbent.

The association between the initial concentration of Pb(ii), which ranged from 40 to 200 mg L^−1^, and the removal percentage of Pb(ii) is shown in Fig. 7. A regulated pH of 5, an adsorbent dosage of 0.03 g L^−1^, and a contact time of 110 min were used for this analysis. As the initial concentration increased from 40 to 200 mg L^−1^, the Pb(ii) removal percentage fell. This tendency was related to the reduced availability of adsorption sites on the surface of the adsorbent. The elimination efficiency was 99.84% at a Pb(ii) concentration of 40 mg L^−1^. Pb(ii) could migrate quickly toward the surface of the nano-HAp adsorbent at 200 mg L^−1^ due to an increase in the pressure differential between the solution and the adsorbent surface. This promoted collision and interaction with the adsorbent's active sites. However, the adsorbent became saturated at high concentrations after adsorbing Pb(ii), which resulted in a buildup of positive charges. This resulted in electrostatic repulsion, which prevented new metal ions from adhering and lowered the removal efficiency by 52.27%.

#### 3.6.3. Effect of the adsorbent dose

**Fig. 8:**
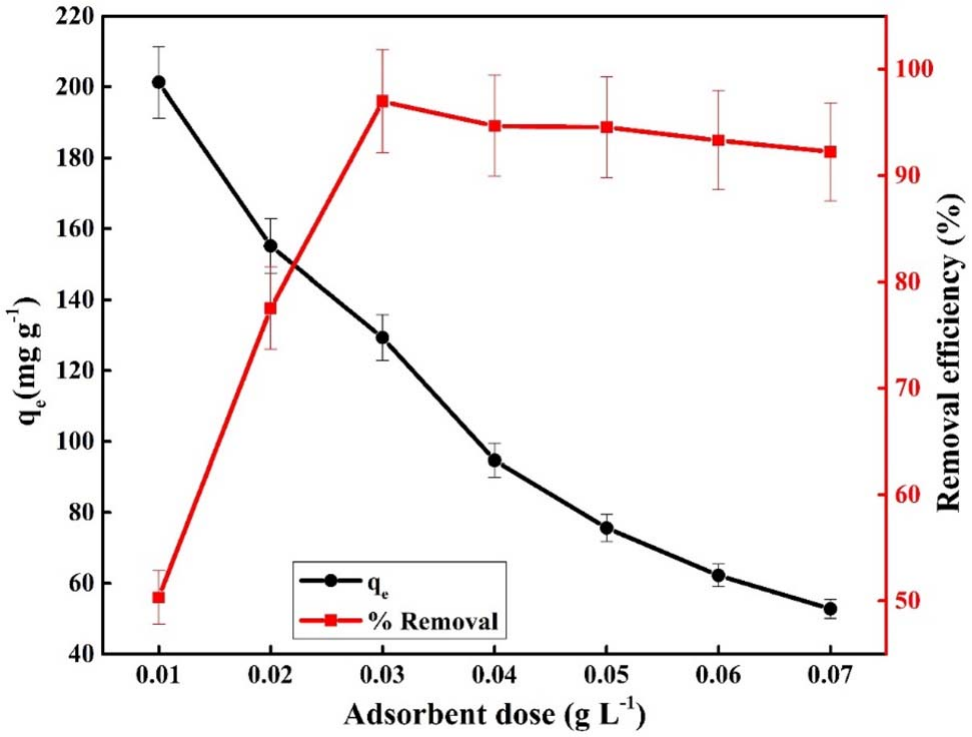
Effect of the adsorbent dose on the removal efficiency of Pb(ii) by the nano-HAp adsorbent.

The effect of changing the nano-HAp adsorbent dose from 0.01 g L^−1^ to 0.06 g L^−1^ on the adsorption of Pb(ii) from an aqueous solution while maintaining a constant initial Pb(ii) concentration of 40 mg L^−1^, pH of 5, and contact time of 110 min is shown in Fig. 8. As a result of the adsorbent surface's high surface area and availability of active empty sites, the percentage removal of Pb(ii) increased from 50.32% to 96.99%, and the adsorbent dose increased from 0.01 g L^−1^ to 0.03 g L^−1^. When the adsorbent dose was increased to 0.06 g L^−1^, the removal efficiency of Pb(ii) declined marginally (93.25%). The adsorbent site being saturated and plateauing may be the cause of the decline in adsorption effectiveness.

#### 3.6.4. Effect of contact time

**Fig. 9:**
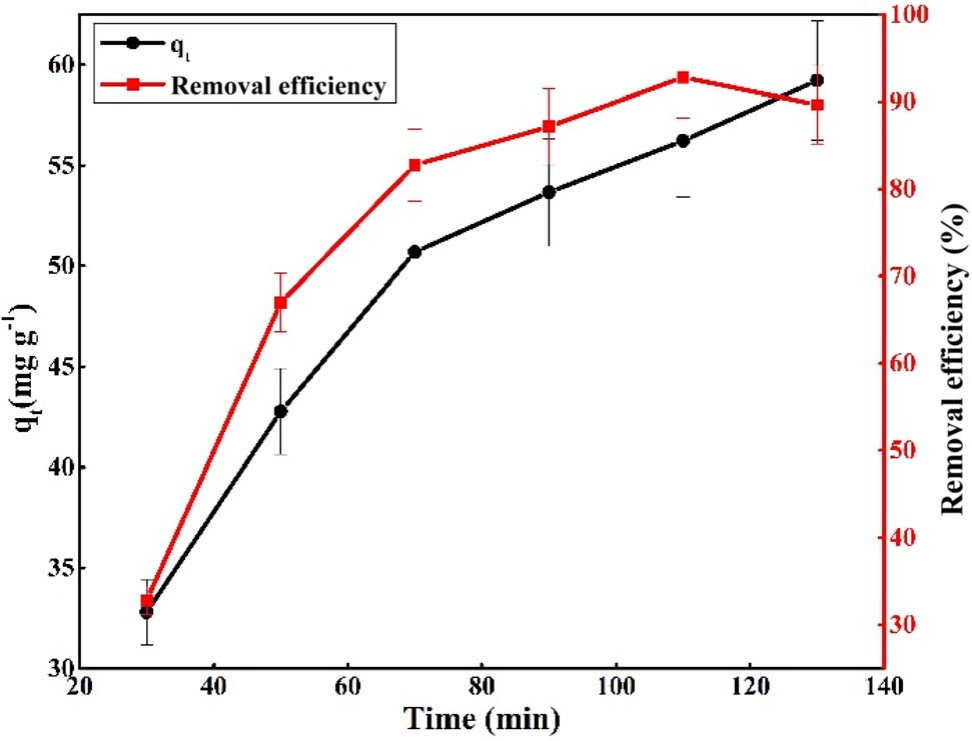
Effect of contact time on the Pb(ii) removal efficiency of the nano-HAp adsorbent.

Fig. 9 displays the percentage removal of Pb(ii) adsorbed throughout a range of contact times, from 30 to 130 min. Based on the findings, it was found that the percentage removal of Pb(ii) increased. The percentage increased sharply from 32.82% to 92.82% as the initial 30 min of contact time increased to 110 min. The large number of active sites available for Pb(ii) delivery explains this remarkable performance. It is possible to draw the conclusion that the removal efficiency improves with contact time, reaching equilibrium after 110 min. Increasing the contact time over 110 min did not improve the effectiveness of Pb(ii) elimination.

### 3.7. Adsorption isotherms

**Fig. 10:**
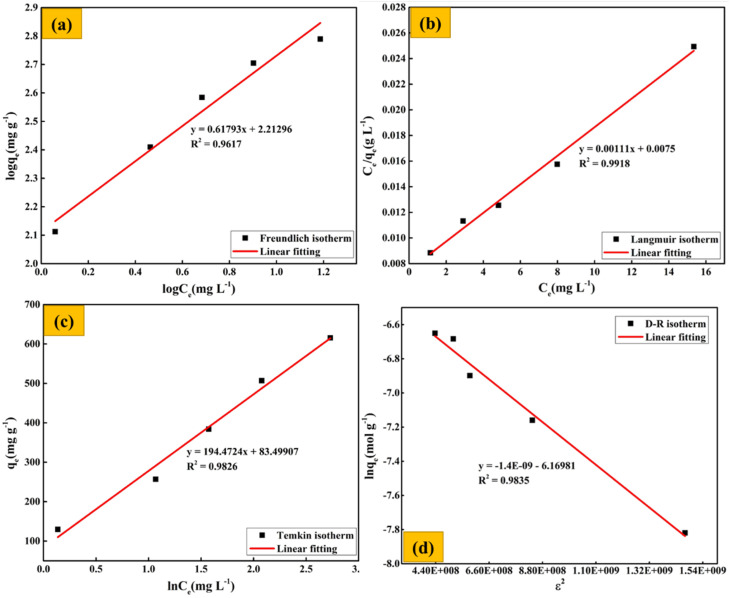
Freundlich (a), Langmuir (b), (c) Temkin, and (d) D–R isotherms in their linear forms.

Fig. 10a–d illustrates the correlation between equilibrium results and four isotherm models, such as the Freundlich, Langmuir, Temkin and Dubinin–Radushkevich (D–R), highlighting their respective fitting accuracies. Table 1 displays the corresponding model parameters. These models were used to describe the interaction between the adsorbates and adsorbents. Every model has its parameters that help in understanding the adsorption process, thereby providing insights into the mechanisms and efficiencies of different adsorbent materials.^56^ As shown in Fig. 10b, the Langmuir isotherm model possesses a better fit to the dataset, as indicated by the coefficient of determination (*R*^2^ = 0.9912). Conversely, Freundlich (*R*^2^ = 0.9617) had a lower value than the Langmuir model. The Langmuir isotherm identifies a monolayer homogeneous surface based on adsorption distribution upon an adsorbent surface. Further, the obtained result revealed a preferable maximum adsorption capacity (*q*_max_) of Langmuir (133.33 mg g^−1^), which indicated that the nano-HAp adsorbent had a desirable maximum adsorption capacity towards Pb(ii). The Freundlich isotherm describes adsorption on heterogeneous surfaces and does not assume a fixed number of sites. In the Freundlich isotherm model, the values of *n* and *K*_F_ were obtained from the slope (1/*n*) and intercept (ln *K*_F_) of the plot of ln *q*_e_*versus* ln *C*_e_ (Fig. 10a). The *n* and *K*_F_ values were 1.62 and 8.27 L g^−1^, respectively, indicating chemical adsorption according to the Freundlich isotherm model, while the *K*_L_ and *R*_L_ values of the Langmuir isotherm model were 6.76 and 0.001, respectively. The obtained *R*_L_ value between 0 and 1 indicated that the adsorption of Pb(ii) on the nano-HAp adsorbent was a favorable condition. With a high coefficient of determination (*R*^2^ = 0.9826), the Temkin model parameters shown in Fig. 10c show that the adsorption of various metal ions employing the nano-HAp adsorbent conforms well to the model. Table 1 demonstrates the Temkin constants (*b*_T_ and *A*_T_) for Pb(ii). With a uniform distribution of binding energies, this isotherm indicated that the adsorption heat decreased linearly with increased coverage while taking indirect interactions in the adsorption dynamics into consideration.^57^ Moreover, based on the correlation coefficient (*R*^2^ = 0.9836), the Dubinin–Radushkevich (D–R) isotherm model is compatible with the Langmuir isotherm model. Additionally, since the value of *E* in the D–R model exceeded 21 kJ mol^−1^, it indicated that the adsorption nature was chemisorption (Fig. 10d).^58^

**Table 1:** Parameters of the Freundlich, Langmuir, Temkin, and D–R isotherms

Isotherm model	Parameter	Value	*R* ^2^
Freundlich	*n*	1.62	0.9617
	*K*_F_ (L g^−1^)	8.27	
Langmuir	*q*_m_ (mg g^−1^)	133.33	0.9912
	*K*_L_ (L mg^−1^)	6.76	
	*R*_L_	0.001	
Temkin	*b*_T_ (J mol^−1^)	194.47	0.9826
	*A*_T_ (L mg^−1^)	1.54	
D–R	*q*_m_ (mol g^−1^)	0.0021	0.9835
	*E* (kJ mol^−1^)	21	
	*β* (mol^2^ kJ^−2^)	1.14 × 10^−9^	

### 3.8. Adsorption kinetic models

**Fig. 11:**
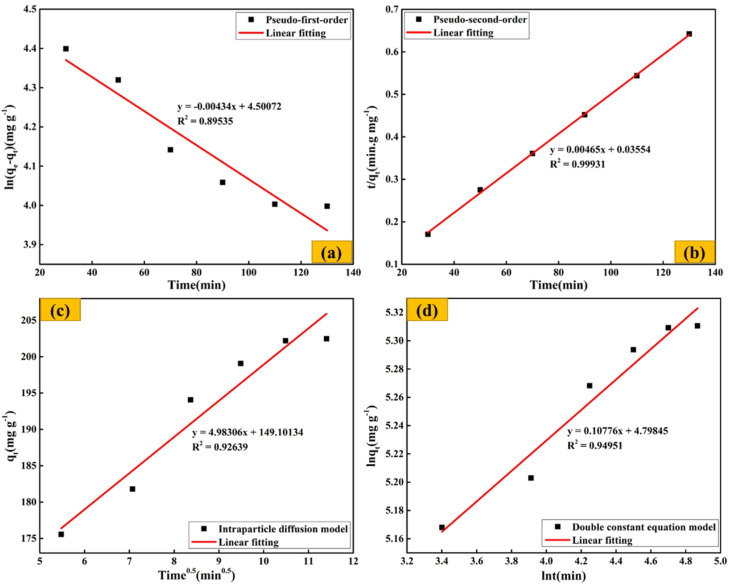
Kinetic plots for the adsorption of Pb(ii) of the (a) pseudo-first-order, (b) pseudo-second-order, (c) intraparticle diffusion, and (d) double constant equation models.

The linear kinetic PFO, PSO, IPD, and DCE models are displayed in Fig. 11a–d and Table 2. A plot of ln(*q*_e_–*q*_t_) against time (*t*) for PFO is shown in Fig. 11a. The PFO model usually describes physisorption, in which Pb(ii) tangentially binds to the adsorbent; however, the low *R*^2^ (0.8954) indicates that it only partially explains adsorption dynamics. A strong fit to the data was indicated by the PSO model's coefficient of regression (*R*^2^) value of 0.9993, which improved the predicted accuracy (Fig. 11b). This high match indicated that the rate-limiting phase in the adsorption process was Pb(ii) chemisorption onto the nano-HAp adsorbent surface.^59^ The PSO model suggested that the main mechanism of adsorption was chemisorption, which was the process by which molecules of the adsorbate formed a chemical bond with the surface of the nano-HAp adsorbent. Fig. 11c shows the IPD curve of the Pb(ii) adsorption onto the nano-HAp adsorbent. The IPD model's high (*R*^2^ = 0.9264) value implies that, in a multi-step process that also involves adsorption, intra-particle diffusion is the main rate-controlling phase. Furthermore, the existence of boundary layer effects and a non-zero intercept (*C* = 149.10 mmol g^−1^) suggested that intra-particle diffusion and surface adsorption took place simultaneously. As shown in Fig. 11d, ln *q*_t_ was plotted against ln *t* according to the double-constant equation model, with an *R*^2^ of 0.9495, indicating a good fit for the adsorption process. As a result, chemisorption drove the adsorption of Pb(ii) on the nano-HAp adsorbent, mostly creating a monolayer on a somewhat heterogeneous surface. The Langmuir and PSO models together showed that the nano-HAp adsorbent worked very well for this purpose, confirming their potential for industrial use in wastewater treatment and environmental remediation.

**Table 2:** Parameters of the pseudo-first-order, pseudo-second-order, intraparticle diffusion, and double-constant equation kinetic models

Kinetic model	Parameter	Value	*R* ^2^
Pseudo-first-order	*q*_e_ exp (mg g^−1^)	256.97	0.8954
	*q*_e_ calc (mg g^−1^)	90.08	
	*k*_1_ (min^−1^)	0.0043	
Pseudo-second-order	*q*_e_ calc (mg g^−1^)	215.05	0.9993
	*k*_2_ (g mg^−1^ min^−1^)	0.001	
Intraparticle diffusion	*k*_i_ (mg g^−1^ min^−0.5^)	4.98	0.9264
Double-constant equation	*A*	121.32	0.9495
	*B*	0.11	

### 3.9. Thermodynamic analysis

**Fig. 12:**
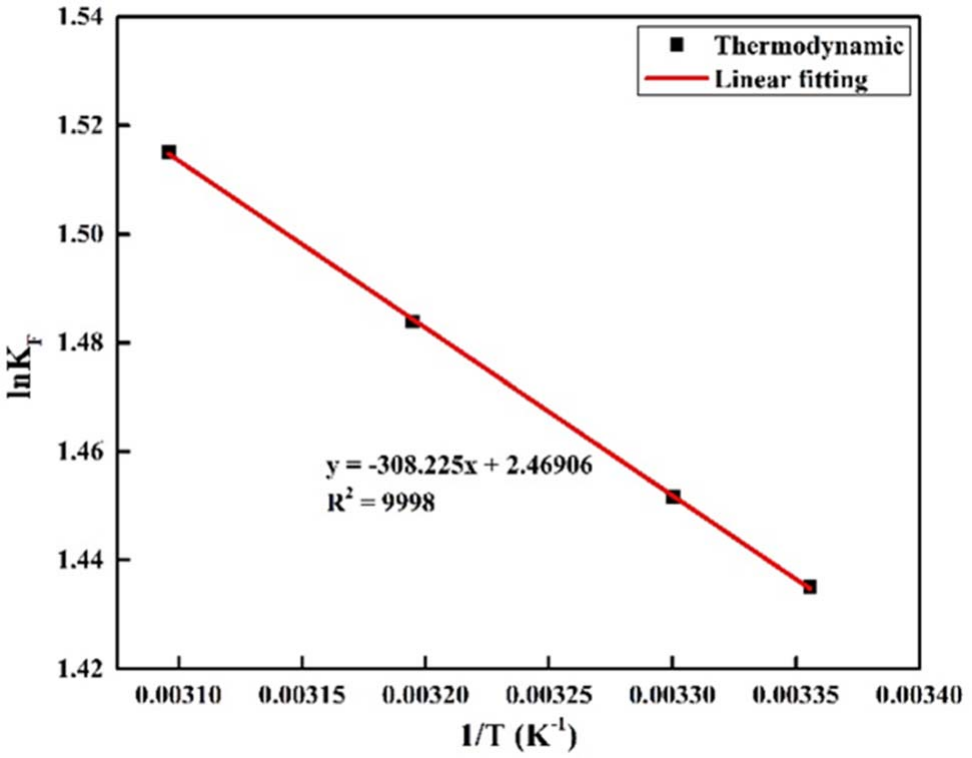
Plot of 1/*T vs.* ln *K*_F_ to determine the thermodynamic parameters for Pb(ii) adsorption onto the nano-HAp adsorbent.

Adsorption thermodynamic analysis was performed at optimum parameters (pH 5, initial Pb(ii) concentration of 40 mg L^−1^, dose of 0.03 g L^−1^ and time of 110 min), with varying the temperature from 298 to 323 K, to analyze the irregularity and spontaneity of Pb(ii) on the nano-HAp adsorbent. Table 3 demonstrates the detailed information of thermodynamic parameters, and Fig. 12 displays the plot of ln *K*_F_ against 1/*T* for further understanding. The enthalpy change (Δ*H*) and entropy change (Δ*S*) were determined from the slope and intercept of the linear graph. The positive Δ*H* value of 2.56 kJ mol^−1^ indicated the endothermic nature of the process, showing that higher temperatures favor Pb(ii) adsorption. While negative Δ*G* confirmed that the Pb(ii) adsorption process was spontaneous and favorable, the Δ*G* values decreased with increasing temperature, indicating that the adsorption of Pb(ii) onto the HAp nanoparticle adsorbent became more favorable at elevated temperatures, reflecting an increase in the driving force for Pb(ii) adsorption. Additionally, the positive Δ*S* value of 20.53 J K^−1^ mol^−1^ indicated an increased randomness inside the system at the interface between the adsorbent and adsorbate.^60^

**Table 3:** Thermodynamic parameters for the adsorption of Pb(ii) onto the nano-HAp adsorbent

Adsorbent	Temperature (K)	*K*_F_	Δ*G* (kJ mol^−1^)	Δ*H* (kJ mol^−1^)	Δ*S* (J K^−1^ mol^−1^)	*R* ^2^
HAp nanoparticles	298	4.20	−3.56	2.56	20.53	0.9998
	303	4.27	−3.66			
	313	4.41	−3.86			
	323	4.55	−4.07			

### 3.10. Adsorption mechanism

**Fig. 13:**
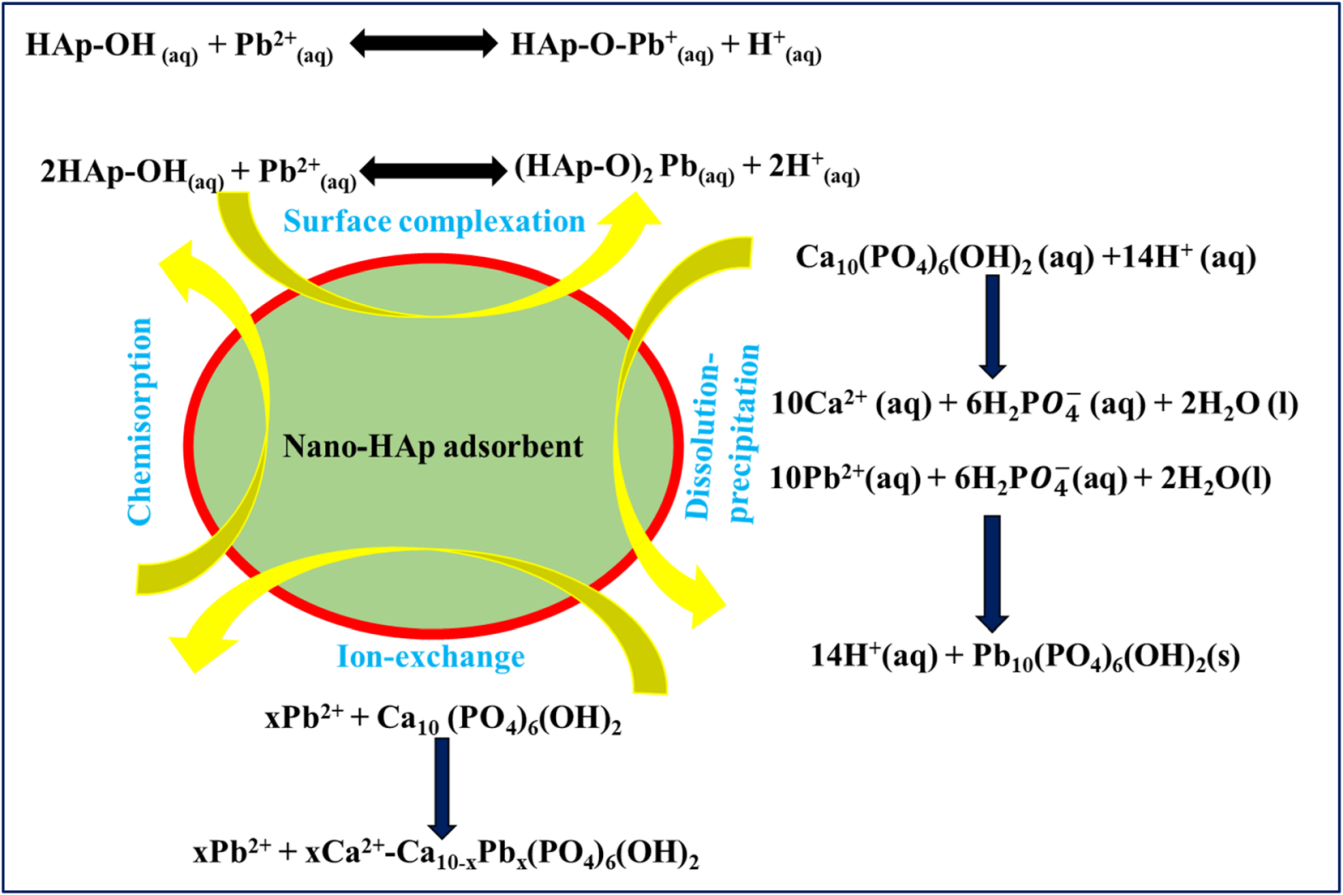
Possible adsorption mechanism of Pb(ii) onto the nano-HAp adsorbent.

To understand the adsorption mechanistic pathways between Pb(ii) and the nano-HAp adsorbent in this study, the XRD, FTIR, and SEM techniques were used. Fig. 1a and b display the XRD patterns of the nano-HAp adsorbent before and after adsorption. As can be seen from Fig. 1b, two new sharp and intense peaks emerged at 2*θ* of around 18.21° and 19.72° after adsorption. This observation suggested that Pb(ii) was successfully adsorbed onto the nano-HAp adsorbent surface. Similarly, slight changes in the peak position were observed. This revealed that the dissolution reaction was involved in the adsorption process. Furthermore, in the FTIR spectra presented in Fig. 2a and d, peak shifts from 3443 cm^−1^ to 3408 cm^−1^, 864 to 814 cm^−1^, and 730 cm^−1^ to 737 cm^−1^ were observed. Moreover, the disappearance of absorption bands at 1463 cm^−1^ and 596 cm^−1^ and the presence of three new peaks, such as 2400 cm^−1^, 2076 cm^−1^, and 1703 cm^−1^, were further evidence of adsorbing Pb(ii) by the nano-HAp adsorbent. The increase in the peak intensity of 1336 cm^−1^ after adsorbing Pb(ii) and the shift in the wavenumber by 49 cm^−1^ indicated that Pb(ii) adsorbed onto the nano-HAp adsorbent surface. Fig. 3c shows the SEM image of the nano-HAp adsorbent after Pb(ii) adsorption and the surface became smoother, indicating that the adsorption sites of the nano-HAp adsorbent successfully interacted with Pb(ii) and generated stable secondary byproducts attached to them. Additionally, the complexation process involving PO_4_^3−^, OH^−^, and Pb(ii) would result in the formation of functional groups, such as PbOH^+^, Pb_2_(OH)^3+^, and Pb(OH)_2_ precipitates and hydroxide complexes, according to pH and pH_pzc_ results. Furthermore, the adsorption was consistent with the Langmuir model (Fig. 10b), indicating monolayer adsorption on a homogeneous surface, according to isotherm investigations. According to the D–R isotherm model, the value of *E* was 21 kJ mol^−1^, suggesting that the adsorption nature was chemisorption (Fig. 10d). Therefore, possible adsorption mechanisms include dissolution-precipitation, ion-exchange, surface complexation, and chemisorption (Fig. 13).^61–66^

### 3.11. Reusability study

**Fig. 14:**
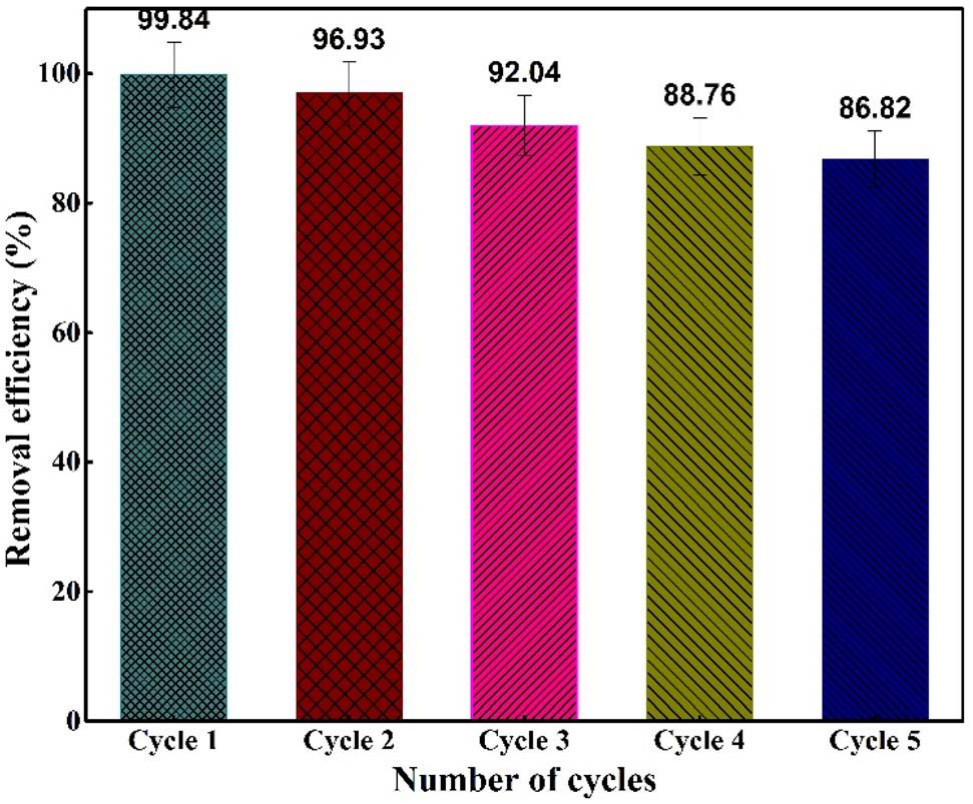
Reusability of the nano-HAp adsorbent for five cycles of Pb(ii) removal.

The regeneration and reusability of the adsorbent are necessary to minimize costs and the expenses associated with producing new adsorbent.^67^ In this study, five adsorption–desorption cycles were performed to evaluate the reusability of the nano-HAp adsorbent. As shown in Fig. 14, the adsorption efficiency over the first cycle was found to be 99.84%. After five consecutive cycles, the adsorption efficiencies decreased to 86.82%. The drop in the adsorption performance may be attributed to the filling of the Pb(ii) residual on the adsorbent surface active sites and a decline in the surface area, which hindered the desorption of Pb(ii) chemically. However, the results illustrated the preferable adsorptive performance of the nano-HAp adsorbent, as it continued to absorb above 86% throughout five successive cycles. Therefore, the prepared nano-HAp adsorbent has significant wastewater-treatment potential due to its eco-friendliness and remarkable reusability performance.

### 3.12. Comparison of the present work with other adsorbents

The maximum monolayer adsorption capacity of the nano-HAp adsorbent for the removal of Pb(ii) was compared with those of other adsorbents reported in the literature. Table 4 shows the evaluation of the feasibility of the adsorbent and a comparison of the adsorption capacity with those of other adsorbents. The table shows that the nano-HAp adsorbent has the highest monolayer adsorption capacity (133.33 mg g^−1^) among all the adsorbents.

**Table 4:** Comparison of the Pb(ii) adsorption capacity onto the adsorbent with that of previously published works

Adsorbent	Factor				*q*_max_ (mg g^−1^)	Removal (%)	Ref.
	pH	Pb(ii) (mg L^−1^)	Dose (g)	Time (min)			
Magnetic bentonite	5	200	10	90	80.40	98.90	68
Fe_3_O_4_@PVBC	5.88	46.51	0.1741	108.21	129.65	97.07	69
MCMC PEI	4.5	50	0.52	180	124	—	70
Alkali-modified wheat bran	4	100	0.15	120	25.32	81.74	71
HMp and Mg0.1-HMp	5	100	2	360	303.03 and 312.50	98.68	72
Nano-HAp	5	40	0.03	110	133.33	99.84	This work

## 4. Conclusion

In this study, cow bone waste was utilized as a precursor for the preparation of the nano-HAp adsorbent *via* a simple ultrasonication-assisted hydrothermal method, followed by calcination. The XRD, FTIR, SEM, BET, and pH_pzc_ analyses were used to characterize the physicochemical characteristics of the nano-HAp adsorbent. The adsorption process was investigated using four parameters, such as pH (2–9), initial Pb(ii) concentration (40–200 mg L^−1^), adsorbent dose (0.01–0.07 g L^−1^), and contact time (30–130 min). From the result, it was found that the optimum adsorption efficiency and adsorption capacity were reached at a pH of 5, initial Pb(ii) concentration of 40 mg L^−1^, adsorbent dose of 0.03 g L^−1^, and contact time of 110 min. The adsorption process was best fitted with the pseudo-second-order kinetic model and Langmuir isotherm model, with a maximum adsorption capacity of 133.33 mg g^−1^. This confirms that the Pb(ii) removal process involves chemisorption and monolayer adsorption onto a homogeneous surface. Furthermore, thermodynamic studies revealed that the adsorption of Pb(ii) was a spontaneous and endothermic process, according to Δ*G* and Δ*H* values. Moreover, the possible adsorption mechanisms were ion-exchange, dissolution-precipitation, surface complexation, and chemisorption. After five reuse cycles, the removal efficiency was higher than 86%. Overall, the prepared nano-HAp adsorbent can be considered a promising material for removing Pb(ii) from aqueous solutions in a cost-effective and efficient manner.

## Author contributions

Endrias Adane Bekele: writing – original draft, writing – review and editing, visualization, validation, investigation, resources, methodology, formal analysis, data curation, conceptualization. Hailemariam Assefa Korsa: writing – original draft, methodology, investigation, formal analysis, data curation, conceptualization. Yiene Molla Desalegn: writing – review and editing, visualization, investigation, software. Andarge Ayele Adem: writing – review and editing, supervision, data curation.

## Conflicts of interest

The authors declare that they have no known competing financial interests or personal relationships that could have appeared to influence the work reported in this paper.

## Data Availability

The datasets generated and analyzed during the current study are available from the corresponding author on reasonable request.
